# Prognostic Value of Conventional Echocardiographic Parameters of Right Ventricular Dysfunction in a Real-World Cohort of Patients With Acute Pulmonary Embolism

**DOI:** 10.7759/cureus.116990

**Published:** 2026-09-27

**Authors:** Mohit Bhagwati, Rahul Mehrotra, Amitabh Yaduvanshi, Vikas Kataria, Mohan Bhargava, Kewal Kishan Talwar, Pankti Shah, Pratyush Kumar, Kumar Abhishek

**Affiliations:** 1 Cardiology, Holy Family Hospital, New Delhi, IND; 2 Cardiology, Artemis Hospitals, New Delhi, IND; 3 Cardiology, Max Super Speciality Hospital, New Delhi, IND; 4 Cardiology, Max Healthcare Institute Limited, New Delhi, IND; 5 Internal Medicine, Centerpoint Medical Center, Independence, USA

**Keywords:** echocardiography, in-hospital mortality, pesi score, prognosis, pulmonary embolism, right ventricular dysfunction, rv fractional area change, tapse

## Abstract

Background: Right ventricular (RV) dysfunction is a known marker of adverse outcomes in acute pulmonary embolism (PE). However, the prognostic utility of individual echocardiographic parameters in clinically heterogeneous patient populations remains a matter of debate. This study aimed to evaluate the association of baseline echocardiographic parameters of RV dysfunction with the Pulmonary Embolism Severity Index (PESI) score and in-hospital mortality.

Methods: We prospectively enrolled 32 consecutive patients with computed tomography-confirmed acute PE. A comprehensive transthoracic echocardiogram was performed within 12 hours of admission. Parameters including tricuspid annular plane systolic excursion (TAPSE), RV fractional area change (RV FAC), tricuspid annular systolic velocity (S'), and RV myocardial performance index (MPI) were measured. The PESI score was calculated for all patients. No a priori power calculation was performed, and analyses were exploratory; significance was judged against a Bonferroni-corrected threshold for the systolic parameters tested.

Results: The mean age was 55.4 ± 18.0 years, and the mean PESI score was 115.2 ± 40.5, indicating a high-risk cohort (59.4% in PESI classes IV-V). In-hospital mortality was 12.5%. Baseline TAPSE, S', and RV FAC showed no significant correlation with the continuous PESI score (p = 0.084, p = 0.235, and p = 0.637, respectively) and no significant association with in-hospital mortality (p > 0.05 for all). Abnormal RV FAC (<35%) was associated with PESI class distribution (χ²(4) = 11.08, p = 0.026, Cramér's V = 0.59) but did not retain significance after correction for multiple testing.

Conclusions: In this small, prospective cohort of patients with acute PE and a high burden of comorbidities, conventional echocardiographic parameters of RV systolic function, including TAPSE and S', were not significantly associated with the PESI score or in-hospital mortality, and the single positive association (RV FAC with PESI class) did not survive correction for multiple testing. Given the limited sample and few events, these exploratory findings are hypothesis-generating and are consistent with an adjunctive - rather than stand-alone - role for echocardiographic RV assessment in risk stratification.

## Introduction

Acute pulmonary embolism (PE) is the third leading cause of cardiovascular death globally, presenting a wide clinical spectrum from asymptomatic incidental findings to cardiogenic shock and sudden death [1-3]. The central pathophysiological event in acute PE is obstruction of the pulmonary arterial tree, which precipitates a rapid increase in pulmonary vascular resistance and a sudden, severe afterload challenge to the right ventricle (RV). The thin-walled, compliant RV is ill-equipped to handle this acute pressure overload, leading to a cascade of RV dilation, contractile dysfunction, interventricular septal bowing that impairs left ventricular filling, and, ultimately, RV failure - the principal mechanism of death in severe PE [4-6].

Given the pivotal role of RV function in determining short-term outcomes, risk stratification is a cornerstone of modern PE management. Clinical prediction rules, most notably the Pulmonary Embolism Severity Index (PESI) and its simplified version (sPESI), have been validated to estimate 30-day mortality based on clinical variables [7,8]. These scores are highly effective at identifying low-risk patients who may be candidates for outpatient management. However, a significant limitation of these clinical scores is their lack of imaging or biomarker data, which are crucial for assessing the hemodynamic impact of the PE. Consequently, international guidelines from the European Society of Cardiology (ESC) and the American Heart Association (AHA) recommend further stratification for normotensive patients, particularly those classified as intermediate-risk, using evidence of RV dysfunction (RVD) on imaging and/or elevated cardiac biomarkers [9,10].

Risk stratification carries direct therapeutic implications. The PESI and sPESI reliably identify low-risk patients who may be eligible for early discharge or outpatient anticoagulation, whereas higher-risk classes warrant inpatient monitoring and, in hemodynamically unstable patients, consideration of systemic thrombolysis or catheter-directed reperfusion. Anticoagulation remains the cornerstone of both acute treatment and secondary prevention, with direct oral anticoagulants now first-line in most patients; adequate intensity and duration of anticoagulation reduce the risk of recurrence and of chronic sequelae such as chronic thromboembolic pulmonary hypertension. When anticoagulation fails or is contraindicated because of a high bleeding risk, inferior vena cava filters provide a mechanical alternative, although their clinical efficacy and adverse-event profile remain incompletely defined [11]. Particular care is required in specific populations - including patients with active malignancy or with inflammatory thrombotic conditions, such as Behçet's syndrome - in whom thrombosis reflects an underlying thromboinflammatory state and the anticoagulation strategy must be individualized against competing bleeding and disease-specific risks [12].

Transthoracic echocardiography (TTE) is a non-invasive, readily available, and powerful imaging modality for the hemodynamic assessment of patients with suspected or confirmed PE [13-16]. Its utility in the emergency setting is well-established, as the absence of echocardiographic signs of RVD can help exclude PE as the cause of hemodynamic instability. Multiple meta-analyses have confirmed that the presence of echocardiographic RVD is associated with an increased risk of adverse outcomes [17-20]. A comprehensive 2022 meta-analysis reported that in hemodynamically stable patients, RVD was associated with an odds ratio (OR) of 2.00 for all-cause mortality and an OR of 4.01 for PE-related mortality [18].

This knowledge gap is clinically important: the prognostic performance of echocardiographic parameters may depend on the baseline clinical risk profile of the cohort. Significant comorbidities, such as active malignancy, may confound the association between RVD and PE-related outcomes, because competing causes of death can attenuate the signal from RV-specific markers.

This study therefore prospectively evaluated conventional two-dimensional echocardiographic parameters of RV systolic function in a real-world cohort with computed tomography pulmonary angiogram (CTPA)-confirmed acute PE. The primary aims were (1) to assess the correlation of baseline tricuspid annular plane systolic excursion (TAPSE), tricuspid annular systolic velocity (S'), and RV fractional area change (FAC) with the continuous PESI score and (2) to assess the association of these parameters, as both continuous and dichotomous variables, with all-cause in-hospital mortality. Comparisons of dichotomized echo parameters with PESI class (I-V) were exploratory. We hypothesized that TAPSE, S', and RV FAC would correlate with the continuous PESI score and would differ between in-hospital survivors and non-survivors.

## Materials and methods

Study design and population

This prospective, non-randomized, observational study was conducted in the Department of Cardiology at Max Superspeciality Hospital, New Delhi, India, over one year from February 2018 to January 2019. The study enrolled 32 consecutive adult patients (age >18 years) with a diagnosis of acute PE confirmed by CTPA. Patients with a prior history of PE were excluded. Thirty-two patients met eligibility criteria and were enrolled. Primary echocardiographic parameters (TAPSE, RV FAC, and S') were complete in all enrolled patients. The study protocol was approved by the Institutional Ethics Committee of Max Super Speciality Hospital, Saket, New Delhi, India (approval number: TS/MSSH/SKT-2/CARDIO/IEC/18-19), and all participants provided written informed consent.

Clinical and laboratory assessment

Upon admission, baseline data were collected using a standardized case report form (CRF), including patient demographics and clinical symptoms. Comorbidities recorded on the CRF included a history of active or prior malignancy, chronic heart failure, chronic lung disease, systemic hypertension, diabetes mellitus, coronary artery disease, chronic kidney disease, and prior venous thromboembolism (a prior history of pulmonary embolism was an exclusion criterion). The standardized physical assessment comprised heart rate, systolic and diastolic blood pressure, respiratory rate, peripheral oxygen saturation, and temperature, together with a focused cardiopulmonary examination for signs of RV strain or failure (elevated jugular venous pressure, an accentuated pulmonary component of the second heart sound, a left parasternal heave, a tricuspid regurgitation murmur, and peripheral edema), pulmonary auscultation, and examination of the lower limbs for signs of deep vein thrombosis. For each patient, the PESI score was calculated based on the 11 established clinical variables to stratify 30-day mortality risk, as originally derived and validated by Aujesky et al. [7]. The PESI is a freely available clinical prediction rule that is not subject to licensing or permission requirements; the score was calculated by the investigators from variables recorded on the study CRF, and no part of the original scoring instrument was reproduced in this report. Blood samples were obtained on admission for the measurement of cardiac biomarkers, including D-dimer, troponin I, creatine kinase-MB (CK-MB), and N-terminal pro-B-type natriuretic peptide (NT-proBNP). Acute PE therapy, including anticoagulation, reperfusion, inferior vena cava filter placement, and hemodynamic support, was determined by the treating team and was not collected as a study endpoint.

Echocardiographic protocol

All patients underwent a comprehensive transthoracic echocardiogram within 12 hours of admission, performed by experienced cardiologists using either a Philips Epiq 7 or a Philips CX 50 portable ultrasound system (Philips Healthcare, Andover, MA). All measurements were performed in accordance with the 2010 guidelines from the American Society of Echocardiography (ASE) for the assessment of the right heart [21]. Measurements were not repeated for formal intra- or interobserver variability analysis. RV systolic function was quantified using multiple parameters: TAPSE, measured from the apical four-chamber view using an M-mode cursor placed through the lateral tricuspid annulus; RV FAC, calculated from the apical four-chamber view as 100 × (end-diastolic area − end-systolic area)/end-diastolic area, with the RV endocardium traced in both diastole and systole; S', measured using pulsed-wave tissue Doppler imaging at the lateral tricuspid annulus; and the RV myocardial performance index (MPI, or Tei index), calculated using both the conventional pulsed Doppler and the tissue Doppler methods.

RV pressure overload was estimated from the peak tricuspid regurgitation jet velocity (TR Vmax) by continuous-wave Doppler, and pulmonary artery systolic pressure (PASP) was estimated using the simplified Bernoulli equation plus estimated right atrial pressure. RV diastolic function was assessed using pulsed-wave Doppler of the tricuspid inflow (E and A velocities) and tissue Doppler (E'). McConnell's sign, defined as akinesia of the RV mid-free wall with preserved apical motion, was actively sought in every patient on the apical four-chamber view as part of the standardized protocol rather than noted incidentally. RVD was defined using established abnormal cut-off values for each parameter: TAPSE <1.6 cm, RV FAC <35%, S' <10 cm/s, pulsed Doppler MPI >0.40, and tissue Doppler MPI >0.55. Each parameter was analyzed separately; a composite definition of RVD requiring two or more abnormal parameters was not used.

Study endpoints and statistical analysis

The primary endpoints were (1) the correlation of baseline TAPSE, RV FAC, and S' with the continuous PESI score and (2) the association of these parameters, as both continuous and dichotomous (normal vs. abnormal) variables, with all-cause in-hospital mortality. The association of dichotomized echo parameters with PESI class (I-V) was exploratory.

Statistical analysis was performed using Statistical Product and Service Solutions (SPSS, version 21.0; IBM SPSS Statistics for Windows, Armonk, NY). Continuous variables were presented as mean ± standard deviation (SD), and categorical variables as numbers and percentages. The association between continuous variables was assessed using Pearson or Spearman correlation coefficients. The independent t-test or Mann-Whitney U test was used to compare means between survivor and non-survivor groups. Associations between categorical variables were assessed using the Pearson chi-square test or Fisher's exact test as appropriate. For each categorical comparison we report the test applied, the test statistic with its degrees of freedom, the two-sided p-value, and an effect size (Cramér's V for tables larger than 2×2 and the phi coefficient for 2×2 tables), interpreted using conventional benchmarks of approximately 0.10, 0.30, and 0.50 for small, moderate, and large effects, respectively; odds ratios with 95% confidence intervals are reported for 2×2 comparisons, with the Haldane-Anscombe correction applied where a zero cell precluded direct estimation. Given the small sample, several contingency tables contained expected cell counts below five; for these, the exact test is additionally reported as a sensitivity analysis, and the chi-square result is interpreted with corresponding caution. Because Fisher's exact test does not generate a test statistic or degrees of freedom, these are not reported where that test was applied. No a priori sample-size or power calculation was performed; the study comprised all consecutive eligible patients presenting during the enrollment period, and the sample size was therefore one of convenience. Analyses were exploratory and hypothesis-generating. No multivariable model was fitted because only four in-hospital deaths occurred. All associations are therefore unadjusted. Observational associations do not support causal inference. Because multiple echocardiographic parameters were tested against each endpoint, nominal p-values were not adjusted in the primary analysis; however, results are interpreted in light of multiplicity, and a Bonferroni-corrected threshold (α = 0.05/3 = 0.017 for the three systolic parameters tested) is applied when judging statistical significance. A two-sided nominal p-value of < 0.05 is reported as the unadjusted threshold.

## Results

Patient characteristics

The study cohort consisted of 32 patients with a mean age of 55.4 ± 18.0 years; 20 patients (62.5%) were male. The cohort presented with a significant burden of comorbidities and high clinical risk, as summarized in Table 1. Notably, 28.1% of patients had a history of cancer, and 18.8% had a history of heart failure. The mean PESI score for the cohort was 115.2 ± 40.5, with 19 patients (59.4%) falling into the high-risk PESI class IV or V. All-cause in-hospital mortality occurred in four patients (12.5%). The flow of patients and outcomes stratified by RV FAC is shown in Figure 1.

**Table 1:** Baseline clinical, biomarker, and PESI characteristics of the study cohort (N=32) Values are mean ± SD for continuous variables and n (%) for categorical variables. BP, blood pressure; NT-proBNP, N-terminal pro-B-type natriuretic peptide; PESI, Pulmonary Embolism Severity Index; SD, standard deviation

Characteristic	Value
Demographics	
Age (years), mean ± SD	55.4 ± 18.0
Male sex, n (%)	20 (62.5)
Comorbidities, n (%)	
History of cancer	9 (28.1)
History of heart failure	6 (18.8)
History of chronic lung disease	2 (6.3)
Clinical findings, n (%)	
Pulse ≥110 bpm	26 (81.3)
Systolic BP <100 mmHg	7 (21.9)
Respiratory rate ≥30/min	16 (50.0)
Arterial O_2_ saturation <90%	13 (40.6)
Biomarkers, mean ± SD	
D-dimer (ng/mL)	3003.7 ± 1909.6
Troponin I (ng/mL)	0.2 ± 0.37
NT-proBNP (pg/mL)	3416.0 ± 3411.9
PESI score, n (%)	
Class I	4 (12.5)
Class II	4 (12.5)
Class III	5 (15.6)
Class IV	6 (18.8)
Class V	13 (40.6)

**Figure 1:**
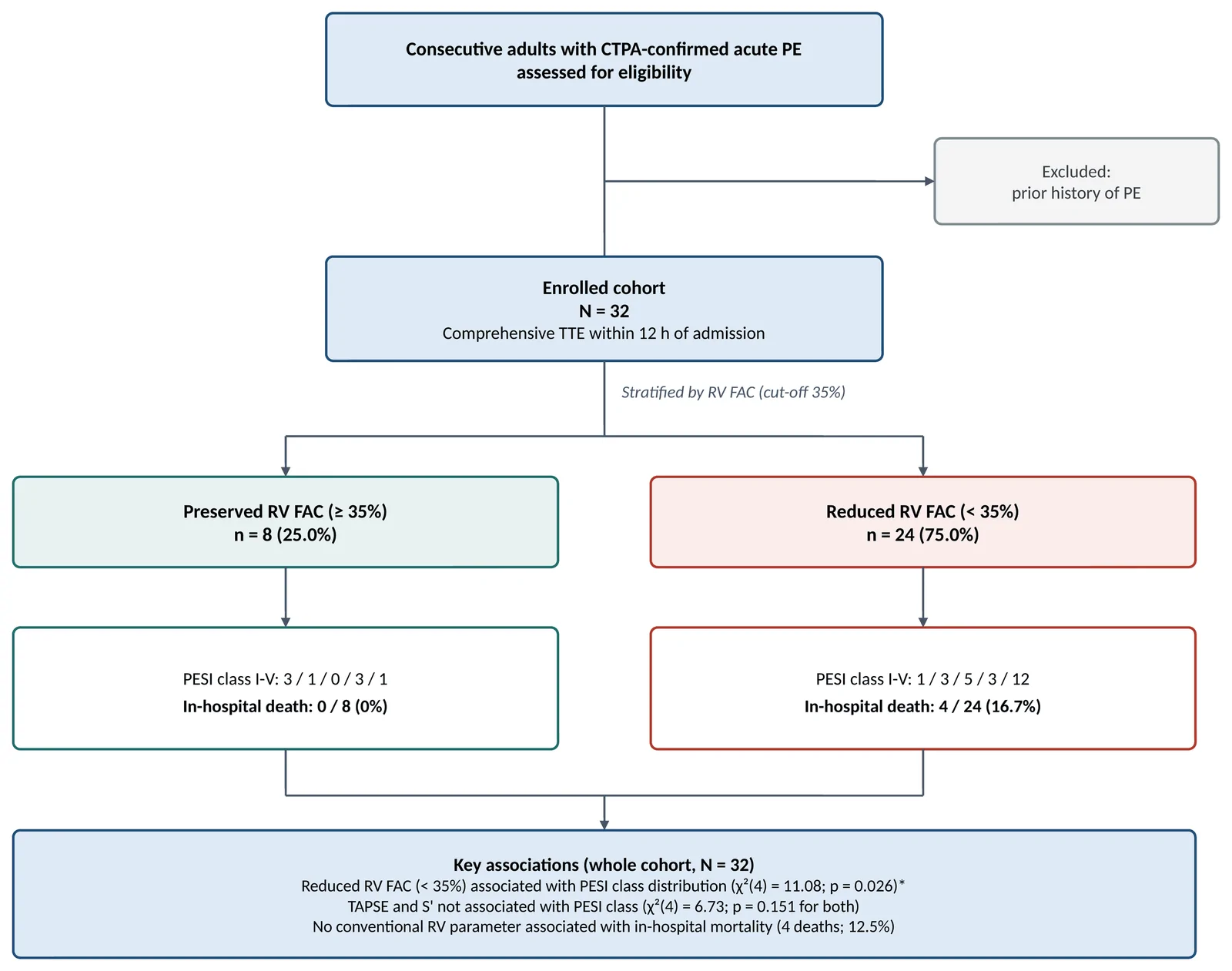
Study flow and outcomes stratified by right ventricular fractional area change Of 32 enrolled patients with computed tomography pulmonary angiogram (CTPA)-confirmed acute PE, eight (25.0%) had preserved RV fractional area change (RV FAC, ≥35%), and 24 (75.0%) had reduced RV FAC (<35%). Reduced RV FAC was associated with Pulmonary Embolism Severity Index (PESI) class distribution (χ²(4) = 11.08, p = 0.026), whereas tricuspid annular plane systolic excursion (TAPSE) and tricuspid annular systolic velocity (S') were associated with neither PESI class nor in-hospital mortality. *The asterisk denotes that this association did not retain statistical significance after Bonferroni correction for the three RV systolic parameters tested (corrected threshold α = 0.05/3 = 0.017); it is therefore reported as exploratory. CTPA, computed tomography pulmonary angiography; PE, pulmonary embolism; PESI, Pulmonary Embolism Severity Index; RV FAC, right ventricular fractional area change; S', tricuspid annular systolic velocity; TAPSE, tricuspid annular plane systolic excursion; TTE, transthoracic echocardiography

Baseline echocardiographic findings

The mean left ventricular ejection fraction was preserved at 58.4 ± 4.8%. Patients exhibited significant evidence of RV pressure overload and dysfunction at baseline, as detailed in Table 2. The mean estimated PASP was 41.4 ± 12.1 mmHg. Based on ASE-recommended cut-offs, RVD was highly prevalent: 75.0% of patients had an abnormal RV FAC (<35%), 56.3% had an abnormal TAPSE (<1.6 cm), and 56.3% had an abnormal S' (<10 cm/s). McConnell's sign was present in 21 patients (65.6%). Representative apical four-chamber stills contrasting preserved and reduced RV FAC are shown in Figure 2.

**Figure 2:**
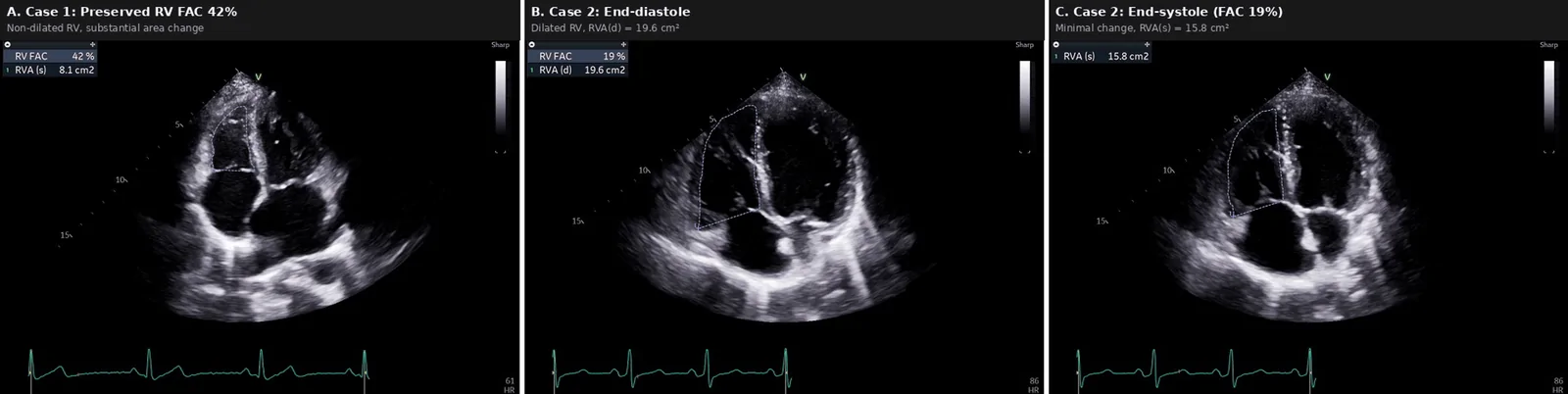
Apical four-chamber 2D echocardiography contrasting preserved and reduced right ventricular fractional area change (RV FAC) (A) Case 1 (preserved RV FAC 42%): non-dilated right ventricle with substantial systolic area change. (B, C) Case 2 (reduced RV FAC 19%): dilated right ventricle showing end-diastolic area 19.6 cm² (B) and end-systolic area 15.8 cm² (C), illustrating minimal area change between diastole and systole.

**Table 2:** Baseline echocardiographic parameters of the study cohort (N=32) Values are mean ± SD. Abnormal cut-offs applied were tricuspid annular plane systolic excursion (TAPSE) <1.6 cm, RV fractional area change (RV FAC) <35%, tricuspid annular systolic velocity (S') <10 cm/s, pulsed Doppler MPI >0.40, and tissue Doppler MPI >0.55. E/A, ratio of early to late diastolic tricuspid inflow velocity; E/E', ratio of early diastolic tricuspid inflow velocity to tissue Doppler annular velocity; LV, left ventricular; LVEF, left ventricular ejection fraction; MPI, myocardial performance index; PASP, pulmonary artery systolic pressure; RV, right ventricular; RV FAC, right ventricular fractional area change; S', tricuspid annular systolic velocity; SD, standard deviation; TAPSE, tricuspid annular plane systolic excursion; TR Vmax, peak tricuspid regurgitation jet velocity

Parameter (mean ± SD)	Value
LV function	
LVEF (%)	58.4 ± 4.8
RV systolic function	
TAPSE (cm)	1.59 ± 0.37
RV FAC (%)	32.3 ± 4.1
S' (cm/s)	9.5 ± 1.7
RV MPI (pulsed Doppler)	0.45 ± 0.06
RV MPI (tissue Doppler)	0.59 ± 0.06
RV diastolic function	
E/A ratio	1.42 ± 0.64
E/E' ratio	7.95 ± 1.25
Hemodynamic estimates	
TR Vmax (m/s)	3.01 ± 0.48
Estimated PASP (mmHg)	41.4 ± 12.1

Association between echocardiographic parameters and the PESI score

There was no statistically significant correlation between the continuous PESI score and the primary echocardiographic measures of RV systolic function at baseline. The correlation coefficient (r) was −0.31 for TAPSE (p = 0.084), −0.216 for S' (p = 0.235), and −0.087 for RV FAC (p = 0.637).

When each echocardiographic parameter was dichotomized (normal vs. abnormal) and cross-tabulated against PESI class (I-V), an abnormal RV FAC (<35%) was associated with PESI class distribution (χ²(4) = 11.08, p=0.026, Cramér's V = 0.59). Among the 24 patients with an abnormal RV FAC, 12 (50.0%) were in class V and one (4.2%) in class I, whereas among the eight patients with a normal RV FAC, one (12.5%) was in class V and three (37.5%) in class I. This association did not retain statistical significance after Bonferroni correction for the three parameters tested (corrected threshold p < 0.017), and the exact test yielded p = 0.017; it is therefore reported as exploratory. Nine of the 10 cells had expected counts below five, so the chi-square approximation is unreliable, and the effect size estimate is imprecise. Neither an abnormal TAPSE (<1.6 cm) nor an abnormal S' (<10 cm/s) was associated with PESI class (both χ²(4) = 6.73, p = 0.151, Cramér's V = 0.46; exact p = 0.164), the two parameters being concordant in all 32 patients.

Association between echocardiographic parameters and in-hospital mortality

There was no statistically significant association between baseline RV systolic function and all-cause in-hospital mortality. The mean baseline TAPSE was 1.61 ± 0.38 cm in survivors versus 1.42 ± 0.34 cm in non-survivors (p = 0.288). Similarly, no significant differences were observed for mean RV FAC (32.5 ± 4.2% vs. 31.0 ± 2.9%; p = 0.488) or mean S' (9.65 ± 1.78 cm/s vs. 8.78 ± 1.29 cm/s; p = 0.351) between the two outcome groups.

Dichotomized RV parameters were likewise not associated with in-hospital mortality. Death occurred in three of 18 patients (16.7%) with an abnormal TAPSE versus one of 14 (7.1%) with a normal TAPSE (Fisher's exact test, p = 0.613; φ = 0.14; OR: 2.60, 95% CI: 0.24-28.15), and in four of 24 patients (16.7%) with an abnormal RV FAC versus none of eight (0%) with a normal RV FAC (Fisher's exact test, p = 0.550; φ = 0.22; OR: 3.73, 95% CI: 0.18-77.18, with the Haldane-Anscombe correction applied for the zero cell). Results for S' were identical to those for TAPSE (Fisher's exact test, p = 0.613; φ = 0.14; OR: 2.60, 95% CI: 0.24-28.15). All three comparisons contained expected cell counts below five, so Fisher's exact test was applied throughout. The confidence intervals are extremely wide, reflecting only four deaths in the cohort, and these analyses were substantially underpowered (see Discussion).

## Discussion

The principal finding of this prospective study is the absence of a statistically significant unadjusted association between conventional echocardiographic markers of RV systolic dysfunction and either the clinical PESI score or in-hospital mortality in a real-world cohort of patients with acute PE and a high burden of comorbidities. TAPSE and S' neither correlated with the PESI score nor predicted in-hospital mortality, and the only positive signal - a nominal association between reduced RV FAC and PESI class distribution - did not survive correction for multiple testing. Rather than identifying any single conventional parameter as a stand-alone prognosticator, these data are most consistent with the current ESC framework, in which echocardiographic evidence of RV dysfunction functions as an adjunct to - not a replacement for - clinical scoring with the PESI and cardiac biomarkers (notably troponin) when stratifying hemodynamically stable patients [9].

The lack of association between TAPSE and adverse outcomes in this cohort stands in contrast to several prominent studies. The work by Pruszczyk et al. in 411 normotensive PE patients established TAPSE as the sole independent echocardiographic predictor of 30-day PE-related mortality or rescue thrombolysis, with a TAPSE ≤15 mm yielding a hazard ratio of 27.9 [22]. A meta-analysis by Coutance et al. likewise reported that abnormal TAPSE was associated with death in all-comers with PE (RR: 2.29) [17]. RV longitudinal strain has more recently emerged as a potentially more sensitive and independent predictor than conventional 2D parameters [23]. One possible explanation for the divergence is the population studied: our cohort carried a substantially higher baseline risk (mean PESI of 115, placing the average patient in class IV, with over 40% in class V) and a 28.1% prevalence of malignancy. That explanation is a hypothesis only. Limited statistical power is an equally important account of the null TAPSE and S' results.

Our results are concordant with a body of work that has questioned the prognostic value of conventional RV parameters in unselected or comorbid populations. In the systematic review and meta-analysis by Prosperi-Porta et al., neither an increased RV/LV ratio nor an abnormal TAPSE was significantly associated with death among hemodynamically stable patients, despite the overall prognostic value of RV dysfunction [18,19]. Similar attenuation of the prognostic signal of single-plane longitudinal indices has been reported in clinically complex cohorts where comorbidities dominate outcomes [24-26]. A likely contributor is that TAPSE and S' capture only the longitudinal component of a geometrically complex chamber and are both load- and angle-dependent, so that, under acute pressure overload and competing systemic illness, they may misclassify global RV performance.

One interpretation of the lack of association is that, in patients with advanced comorbidity, short-term mortality is driven by the underlying disease process, sepsis, or frailty rather than by the hemodynamic insult of the PE, attenuating the prognostic signal of RV-specific markers [25-28]. We emphasize, however, that this study recorded only four deaths and was substantially underpowered to detect an association with mortality; a type II (false-negative) error is at least as plausible an explanation as true attenuation, and the two cannot be distinguished in a cohort of this size. The competing-risk hypothesis should therefore be regarded as one possibility generated by, not established by, these data.

We were unable to perform multivariable adjustment for the principal confounders - active malignancy, heart failure, and age - because only four in-hospital deaths occurred. With fewer than one event per candidate covariate, any multivariable mortality model would be grossly overfitted and uninterpretable; the associations reported here are therefore unadjusted and cannot be disentangled from confounding. This limitation reinforces a cautious, hypothesis-generating reading of the results.

The observation that RV FAC was the only parameter to show even a nominal association with PESI class is mechanistically plausible, even though it did not survive correction for multiplicity. Unlike TAPSE and S', which measure only the longitudinal component of RV contraction, RV FAC provides a more global assessment by incorporating both longitudinal and transverse dimensions [29], and it has shown the strongest correlation with the gold-standard cardiac magnetic resonance measure of RV ejection fraction [30]. A global functional index may therefore reflect the severity of RV compromise under acute pressure overload more robustly than single-plane motion parameters, a hypothesis that warrants testing in larger cohorts.

Limitations

This study has several important limitations. The primary limitation is the small sample size of 32 patients with only four events, which severely restricts statistical power, precludes multivariable analysis, and creates a substantial risk of type II error. No a priori power calculation was undertaken, and the analyses were exploratory; given the number of comparisons, the nominally significant RV FAC association should be regarded as hypothesis-generating. The single-center design may limit generalizability. The analysis did not include advanced techniques, such as RV longitudinal strain, which may be more sensitive and independent predictors of adverse outcomes [23]. Finally, the endpoint was all-cause in-hospital mortality, which, in a cohort with high comorbidity, may not solely reflect PE-related deaths.

## Conclusions

In this small prospective cohort of patients with acute PE and a high burden of comorbidities, conventional 2D echocardiographic parameters of RV systolic function, including TAPSE and S', were not significantly associated with the PESI score or in-hospital mortality, and the only positive signal (reduced RV FAC and PESI class distribution) did not survive correction for multiple testing. Given the small sample and the very small number of events, these findings are hypothesis-generating and must be interpreted cautiously; they should not be taken as evidence that conventional RV parameters lack prognostic value. They are, however, consistent with current guidance that positions echocardiographic RV assessment as an adjunct to clinical scoring and biomarkers rather than as a stand-alone prognostic test, and they underscore the need for adequately powered, multicenter studies - ideally incorporating advanced techniques, such as RV strain - to define the prognostic role of RV imaging in clinically complex PE populations.

## References

[1] Konstantinides SV, Meyer G (2019). The 2019 ESC guidelines on the diagnosis and management of acute pulmonary embolism. Eur Heart J.

[2] Goldhaber SZ, Visani L, De Rosa M (1999). Acute pulmonary embolism: Clinical outcomes in the International Cooperative pulmonary embolism Registry (Icoper). Lancet.

[3] Wendelboe AM, Raskob GE (2016). Global burden of thrombosis: epidemiologic aspects. Circ Res.

[4] Lualdi JC, Goldhaber SZ (1995). Right ventricular dysfunction after acute pulmonary embolism: pathophysiologic factors, detection, and therapeutic implications. Am Heart J.

[5] Matthews JC, McLaughlin V (2008). Acute right ventricular failure in the setting of acute pulmonary embolism or chronic pulmonary hypertension: a detailed review of the pathophysiology, diagnosis, and management. Curr Cardiol Rev.

[6] Pinsky MR (2016). The right ventricle: interaction with the pulmonary circulation. Crit Care.

[7] Aujesky D, Obrosky DS, Stone RA (2005). Derivation and validation of a prognostic model for pulmonary embolism. Am J Respir Crit Care Med.

[8] Jiménez D, Aujesky D, Moores L (2010). Simplification of the pulmonary embolism severity index for prognostication in patients with acute symptomatic pulmonary embolism. Arch Intern Med.

[9] Konstantinides SV, Meyer G, Becattini C (2020). 2019 ESC Guidelines for the diagnosis and management of acute pulmonary embolism developed in collaboration with the European Respiratory Society (ERS). Eur Heart J.

[10] Jaff MR, McMurtry MS, Archer SL (2011). Management of massive and submassive pulmonary embolism, iliofemoral deep vein thrombosis, and chronic thromboembolic pulmonary hypertension: a scientific statement from the American Heart Association. Circulation.

[11] Visconti L, Celi A, Carrozzi L (2024). Inferior vena cava filters: concept review and summary of current guidelines. Vascul Pharmacol.

[12] Casto E, Celi A, Madonna R (2024). Anticoagulation in venous thromboembolism in Behçet's syndrome: friend or foe?. RMD Open.

[13] Hritani R, Alrifai A, Soud M, Moussa Pacha H, Alraies MC (2018). Which patients with pulmonary embolism need echocardiography?. Cleve Clin J Med.

[14] Sanchez O, Trinquart L, Planquette B (2013). Echocardiography and pulmonary embolism severity index have independent prognostic roles in pulmonary embolism. Eur Respir J.

[15] Valerio L, Barco S (2020). Risk stratification of normotensive pulmonary embolism: one more ride on the merry-go-round. Eur Heart J Acute Cardiovasc Care.

[16] Nasser MF, Jabri A, Limaye S (2023). Echocardiographic evaluation of pulmonary embolism: a review. J Am Soc Echocardiogr.

[17] Coutance G, Cauderlier E, Ehtisham J, Hamon M, Hamon M (2011). The prognostic value of markers of right ventricular dysfunction in pulmonary embolism: a meta-analysis. Crit Care.

[18] Prosperi-Porta G, Ronksley P, Kiamanesh O, Solverson K, Motazedian P, Weatherald J (2022). Prognostic value of echocardiography-derived right ventricular dysfunction in haemodynamically stable pulmonary embolism: a systematic review and meta-analysis. Eur Respir Rev.

[19] Sanchez O, Trinquart L, Colombet I, Durieux P, Huisman MV, Chatellier G, Meyer G (2008). Prognostic value of right ventricular dysfunction in patients with haemodynamically stable pulmonary embolism: a systematic review. Eur Heart J.

[20] Becattini C, Agnelli G (2007). Acute pulmonary embolism: risk stratification in the emergency department. Intern Emerg Med.

[21] Rudski LG, Lai WW, Afilalo J (2010). Guidelines for the echocardiographic assessment of the right heart in adults: a report from the American Society of Echocardiography endorsed by the European Association of Echocardiography, a registered branch of the European Society of Cardiology, and the Canadian Society of Echocardiography. J Am Soc Echocardiogr.

[22] Pruszczyk P, Goliszek S, Lichodziejewska B (2014). Prognostic value of echocardiography in normotensive patients with acute pulmonary embolism. JACC Cardiovasc Imaging.

[23] Vitarelli A, Barillà F, Capotosto L, D'Angeli I, Truscelli G, De Maio M, Ashurov R (2014). Right ventricular function in acute pulmonary embolism: a combined assessment by three-dimensional and speckle-tracking echocardiography. J Am Soc Echocardiogr.

[24] Khemasuwan D, Yingchoncharoen T, Tunsupon P, Kusunose K, Moghekar A, Klein A, Tonelli AR (2015). Right ventricular echocardiographic parameters are associated with mortality after acute pulmonary embolism. J Am Soc Echocardiogr.

[25] Park JH, Kim JH, Lee JH, Choi SW, Jeong JO, Seong IW (2012). Evaluation of right ventricular systolic function by the analysis of tricuspid annular motion in patients with acute pulmonary embolism. J Cardiovasc Ultrasound.

[26] Dahhan T, Siddiqui I, Tapson VF (2016). Clinical and echocardiographic predictors of mortality in acute pulmonary embolism. Cardiovasc Ultrasound.

[27] Sánchez D, De Miguel J, Sam A (2011). The effects of cause of death classification on prognostic assessment of patients with pulmonary embolism. J Thromb Haemost.

[28] Ribeiro A, Lindmarker P, Juhlin-Dannfelt A (1997). Echocardiography Doppler in pulmonary embolism: right ventricular dysfunction as a predictor of mortality rate. Am Heart J.

[29] Kaul S, Tei C, Hopkins JM, Shah PM (1984). Assessment of right ventricular function using two-dimensional echocardiography. Am Heart J.

[30] Anavekar NS, Gerson D, Skali H, Kwong RY, Yucel EK, Solomon SD (2007). Two-dimensional assessment of right ventricular function: an echocardiographic-MRI correlative study. Echocardiography.

